# 6-Amino-5-(1-amino-2,2-dicyano­vin­yl)-3,3a,4,5-tetra­hydro-2*H*-indene-4-spiro-1′-cyclo­pentane-3a,7-dicarbonitrile–thio­phene-2-carbaldehyde (1/0.5)

**DOI:** 10.1107/S1600536810034434

**Published:** 2010-09-04

**Authors:** Abdullah M. Asiri, Seik Weng Ng

**Affiliations:** a Department of Chemistry, Faculty of Science, King Abdul Aziz University, PO Box 80203, Jeddah, Saudi Arabia; b Department of Chemistry, University of Malaya, 50603 Kuala Lumpur, Malaysia

## Abstract

In each of the two independent indene-4-spiro­pentane mol­ecules in the asymmetric unit of the title 2:1 adduct, C_19_H_18_N_6_·0.5C_5_H_4_OS, the cyclo­hexene ring adopts a half-chair conformation and the cyclo­pentene and cyclo­pentane rings adopt envelope conformations. The mean plane through the cyclo­hexene/cyclo­pentene fused system is aligned at a dihedral angle of 77.9 (1)° with respect to the mean plane through the cyclo­pentane ring in one mol­ecule and 87.0 (1)° in the other. In the crystal, adjacent indene-4-spiro­pentane mol­ecules are linked by N—H⋯N hydrogen bonds into a three-dimensional network. The spaces within the network are occupied by the thio­phene-2-carbaldehyde mol­ecules. The thio­phene-2-carbaldehyde unit is disordered over two positions of equal occupancy. The crystal studied was found to be a non-morohedral twin with two minor twin components of 18.4 and 9.7%.

## Related literature

For our report of the condensation of cyclopentylidene­malono­nitrile and thiophene-2-carbaldehyde to form 2,5-bis(thienylidene)-1-dicyanomethylene-cyclopentane, a purple-colored compound suitable for application as a dye, see: Asiri (2003 ▶). For a related structure, see: Nesterov & Viltchinskaia (2000 ▶). For the treatment of twinned diffraction data, see: Spek (2009 ▶).
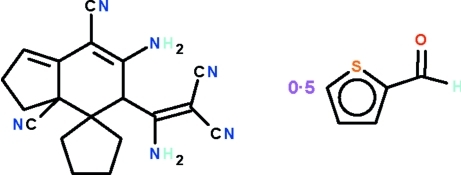


## Experimental

### 

#### Crystal data

C_19_H_18_N_6_·0.5C_5_H_4_OS
*M* *_r_* = 386.46
Triclinic, 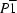

*a* = 12.0181 (14) Å
*b* = 13.8109 (15) Å
*c* = 13.9000 (15) Å
α = 60.703 (1)°
β = 77.841 (2)°
γ = 89.879 (2)°
*V* = 1953.1 (4) Å^3^
*Z* = 4
Mo *K*α radiation
μ = 0.14 mm^−1^
*T* = 100 K
0.45 × 0.05 × 0.05 mm

#### Data collection

Bruker SMART APEX diffractometer
Absorption correction: multi-scan (*SADABS*; Sheldrick, 1996 ▶) *T*_min_ = 0.942, *T*_max_ = 0.993
18784 measured reflections
8916 independent reflections
5141 reflections with *I* > 2σ(*I*)
*R*_int_ = 0.068

#### Refinement

*R*[*F* ^2^ > 2σ(*F* ^2^)] = 0.074
*wR*(*F* ^2^) = 0.216
*S* = 1.02
8916 reflections
571 parameters
94 restraints
H-atom parameters constrained
Δρ_max_ = 0.59 e Å^−3^
Δρ_min_ = −0.50 e Å^−3^

### 

Data collection: *APEX2* (Bruker, 2009 ▶); cell refinement: *SAINT* (Bruker, 2009 ▶); data reduction: *SAINT*; program(s) used to solve structure: *SHELXS97* (Sheldrick, 2008 ▶); program(s) used to refine structure: *SHELXL97* (Sheldrick, 2008 ▶); molecular graphics: *X-SEED* (Barbour, 2001 ▶); software used to prepare material for publication: *publCIF* (Westrip, 2010 ▶).

## Supplementary Material

Crystal structure: contains datablocks global, I. DOI: 10.1107/S1600536810034434/xu5020sup1.cif

Structure factors: contains datablocks I. DOI: 10.1107/S1600536810034434/xu5020Isup2.hkl

Additional supplementary materials:  crystallographic information; 3D view; checkCIF report

## Figures and Tables

**Table 1:** Hydrogen-bond geometry (Å, °)

*D*—H⋯*A*	*D*—H	H⋯*A*	*D*⋯*A*	*D*—H⋯*A*
N2—H2n1⋯N1^i^	0.86	2.23	2.909 (4)	136
N3—H3n1⋯N11^ii^	0.86	2.28	2.970 (4)	138
N3—H3n2⋯N12^ii^	0.86	2.29	3.042 (4)	147
N8—H8n1⋯N7^iii^	0.86	2.45	2.921 (4)	115
N8—H8n2⋯N4^iv^	0.86	2.31	3.006 (4)	138
N9—H9n1⋯N5	0.86	2.39	3.098 (4)	139
N9—H9n2⋯N6	0.86	2.35	3.178 (4)	163

Symmetry codes: (i) 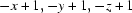; (ii) 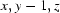; (iii) 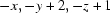; (iv) 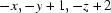.

**Fig. 1.**
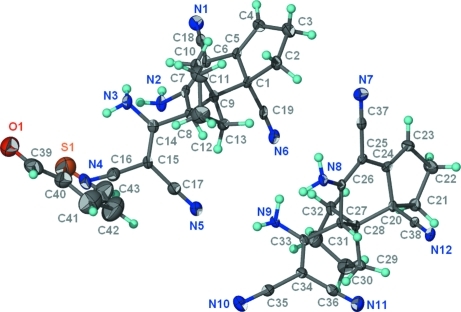
Thermal ellipsoid plot (Barbour, 2001) of the asymmetric unit of the C19H18N6.0.5C5H4OS co-crystal at the 70% probability level; hydrogen atoms are drawn as spheres of arbitrary radius.

## supplementary crystallographic information

### Comment

We have previously reported the condensation of cyclopentylidenemalononitrile and thiophene-2-carbaldehyde to form 2,5-bis(thienylidene)-1-dicyanomethylene-cyclopentane, a purple-colored compound suitable for application as a dye (Asiri, 2003). For reasons that we are not clear of, our attempted synthesis gave only colorless crystals. We examined a plate-like specimen and identified it as 6-amino-5,5,7-tricyano-3,3*a*,4,5- tetrahydro-2*H*-indene-4-spirocyclo-pentane, whose structure has already been reported (Nesterov & Viltchinskaia, 2000). We identified a prismatic specimen as the title 1: 0.5 co-crystal (Scheme I, Fig. 1); the second component is unchanged thiophene-2-carbaldehyde. The first component differs from the reported compound in having an aminodicyanovinyl group (along with a methine hydrogen) in place of the two cyano groups in the 5-position. Additionally, the compound has another cyano group in the 3*a* position.

### Experimental

Cyclopentylidenemalononitrile (0.13 g, 1 mmol) and thiophene-2-carbaldehyde (0.22 g, 2 mmol) were heated in an oil bath for 6 h. Ethanol was added to break up the solid material. The product was collected and recrystallized from acetic acid.

### Refinement

Carbon-bound H-atoms were placed in calculated positions (C—H 0.95 to 0.99 Å) and were included in the refinement in the riding model approximation, with *U*(H) set to 1.2*U*(C). The amino H-atoms were similarly positioned (N–H 0.86 Å) by rotating them; their temperature factors were tied by a factor of 1.2. Modeling the amino group as if it was a methyl group but setting the occupancy factor of one of the three H-atoms to zero gave a satisfactory hydrogen bond scheme except for the H2*n*2 and H3*n*2 atoms, which were 1.82 Å apart.

The thiophene-2-carbaldehyde molecule is disordered over two positions; as the disorder refined to nearly 1:1, the occupancy of each component was set to 0.5. The sulfur–carbon distances were restrained to 1.70±0.01 Å and the oxygen–carbon distances to 1.25±0.01 Å. The excyclic carbon–carbon distances were restrained to 1.50±0.01 Å and the endocyclic ones to 1.35±0.01 Å. All atoms of each component were restrained to lie on a plane. The temperature factors of C41' were set to those of S1, and that of S1' to those of C41 as the pair of atoms are close to each other. The anisotropic temperature factors of the disordered atoms were restrained to be nearly isotropic. The primed carbon atoms were set to those of the unprimed ones; the anisotropic temperature factors of these carbon atoms were restrained to be nearly isotropic.

The structure is a non-meohedrally twinned structure with two minor twin components of 18.5 and 9.7%. The twin domains were identified by the use of *PLATON* (Spek, 2009). The twinned nature led to a somewhat large weighting scheme.

### Crystal data

**Table:**

C_19_H_18_N_6_·0.5C_5_H_4_OS	*V* = 1953.1 (4) Å^3^
*M**_r_* = 386.46	*Z* = 4
Triclinic, *P*1	*F*(000) = 812
Hall symbol: -P 1	*D*_x_ = 1.314 Mg m^−^^3^
*a* = 12.0181 (14) Å	Mo *K*α radiation, λ = 0.71073 Å
*b* = 13.8109 (15) Å	µ = 0.14 mm^−^^1^
*c* = 13.9000 (15) Å	*T* = 100 K
α = 60.703 (1)°	Prism, colorless
β = 77.841 (2)°	0.45 × 0.05 × 0.05 mm
γ = 89.879 (2)°	

### Data collection

**Table:**

Bruker SMART APEX diffractometer	8916 independent reflections
Radiation source: fine-focus sealed tube	5141 reflections with *I* > 2σ(*I*)
graphite	*R*_int_ = 0.068
ω scans	θ_max_ = 27.5°, θ_min_ = 1.7°
Absorption correction: multi-scan (*SADABS*; Sheldrick, 1996)	*h* = −15→15
*T*_min_ = 0.942, *T*_max_ = 0.993	*k* = −17→17
18784 measured reflections	*l* = −18→18

### Refinement

**Table:**

Refinement on *F*^2^	Primary atom site location: structure-invariant direct methods
Least-squares matrix: full	Secondary atom site location: difference Fourier map
*R*[*F*^2^ > 2σ(*F*^2^)] = 0.074	Hydrogen site location: inferred from neighbouring sites
*wR*(*F*^2^) = 0.216	H-atom parameters constrained
*S* = 1.02	*w* = 1/[σ^2^(*F*_o_^2^) + (0.1073*P*)^2^ + 0.3194*P*] where *P* = (*F*_o_^2^ + 2*F*_c_^2^)/3
8916 reflections	(Δ/σ)_max_ = 0.001
571 parameters	Δρ_max_ = 0.59 e Å^−^^3^
94 restraints	Δρ_min_ = −0.50 e Å^−^^3^

### Fractional atomic coordinates and isotropic or equivalent isotropic displacement parameters (Å^2^)

**Table:**

	*x*	*y*	*z*	*U*_iso_*/*U*_eq_	Occ. (<1)
N1	0.4315 (3)	0.5673 (3)	0.3804 (3)	0.0226 (7)	
N2	0.3308 (2)	0.4683 (2)	0.6776 (3)	0.0185 (7)	
H2N1	0.3882	0.4725	0.6262	0.022*	
H2N2	0.2992	0.4002	0.7194	0.022*	
N3	0.0952 (2)	0.3434 (2)	0.8014 (3)	0.0188 (7)	
H3N1	0.0890	0.2827	0.8652	0.023*	
H3N2	0.1512	0.3434	0.7514	0.023*	
N4	0.0331 (3)	0.2262 (2)	1.0946 (3)	0.0251 (7)	
N5	0.1735 (3)	0.5862 (3)	0.9363 (3)	0.0265 (8)	
N6	0.2511 (3)	0.8177 (2)	0.5964 (3)	0.0208 (7)	
N7	0.1317 (3)	1.0660 (3)	0.3768 (3)	0.0232 (7)	
N8	0.0847 (2)	0.9640 (2)	0.6749 (3)	0.0166 (6)	
H8N1	0.0700	0.9439	0.6288	0.020*	
H8N2	0.0894	0.9057	0.7372	0.020*	
N9	0.2564 (2)	0.8396 (2)	0.8127 (3)	0.0167 (6)	
H9N1	0.2118	0.7820	0.8678	0.020*	
H9N2	0.2489	0.8475	0.7491	0.020*	
N10	0.1724 (3)	0.7429 (3)	1.1069 (3)	0.0308 (8)	
N11	0.1138 (3)	1.0999 (3)	0.9271 (3)	0.0283 (8)	
N12	0.2061 (3)	1.3168 (2)	0.5990 (3)	0.0199 (7)	
C1	0.1173 (3)	0.7121 (3)	0.5405 (3)	0.0138 (7)	
C2	0.0404 (3)	0.7911 (3)	0.4653 (3)	0.0182 (8)	
H2A	−0.0368	0.7515	0.4857	0.022*	
H2B	0.0323	0.8573	0.4751	0.022*	
C3	0.1023 (3)	0.8262 (3)	0.3422 (3)	0.0247 (9)	
H3A	0.0473	0.8245	0.2990	0.030*	
H3B	0.1441	0.9023	0.3028	0.030*	
C4	0.1835 (3)	0.7411 (3)	0.3552 (3)	0.0195 (8)	
H4	0.2238	0.7320	0.2941	0.023*	
C5	0.1931 (3)	0.6794 (3)	0.4618 (3)	0.0135 (7)	
C6	0.2654 (3)	0.5934 (3)	0.5125 (3)	0.0133 (7)	
C7	0.2539 (3)	0.5343 (3)	0.6275 (3)	0.0139 (7)	
C8	0.1539 (3)	0.5441 (3)	0.7069 (3)	0.0141 (7)	
H8	0.1863	0.5919	0.7323	0.017*	
C9	0.0552 (3)	0.6065 (3)	0.6523 (3)	0.0149 (7)	
C10	−0.0223 (3)	0.5370 (3)	0.6247 (3)	0.0162 (7)	
H10A	−0.0112	0.5705	0.5416	0.019*	
H10B	−0.0036	0.4590	0.6567	0.019*	
C11	−0.1455 (3)	0.5403 (3)	0.6799 (3)	0.0286 (9)	
H11A	−0.1788	0.6042	0.6259	0.034*	
H11B	−0.1941	0.4702	0.7057	0.034*	
C12	−0.1362 (4)	0.5532 (4)	0.7792 (4)	0.0334 (10)	
H12A	−0.1285	0.4807	0.8445	0.040*	
H12B	−0.2041	0.5839	0.8033	0.040*	
C13	−0.0279 (3)	0.6350 (3)	0.7332 (3)	0.0173 (8)	
H13A	0.0057	0.6258	0.7960	0.021*	
H13B	−0.0453	0.7131	0.6918	0.021*	
C14	0.1147 (3)	0.4307 (3)	0.8139 (3)	0.0150 (7)	
C15	0.1041 (3)	0.4207 (3)	0.9191 (3)	0.0168 (8)	
C16	0.0647 (3)	0.3143 (3)	1.0194 (3)	0.0202 (8)	
C17	0.1406 (3)	0.5114 (3)	0.9310 (3)	0.0195 (8)	
C18	0.3565 (3)	0.5790 (3)	0.4394 (3)	0.0157 (7)	
C19	0.1922 (3)	0.7745 (3)	0.5698 (3)	0.0159 (7)	
C20	0.3623 (3)	1.2092 (3)	0.5421 (3)	0.0133 (7)	
C21	0.4758 (3)	1.2877 (3)	0.4679 (3)	0.0180 (8)	
H21A	0.4808	1.3517	0.4810	0.022*	
H21B	0.5430	1.2466	0.4856	0.022*	
C22	0.4708 (3)	1.3282 (3)	0.3445 (3)	0.0255 (9)	
H22A	0.4475	1.4044	0.3088	0.031*	
H22B	0.5462	1.3284	0.2987	0.031*	
C23	0.3825 (3)	1.2450 (3)	0.3546 (3)	0.0202 (8)	
H23	0.3701	1.2394	0.2921	0.024*	
C24	0.3239 (3)	1.1801 (3)	0.4614 (3)	0.0146 (7)	
C25	0.2275 (3)	1.0928 (3)	0.5128 (3)	0.0134 (7)	
C26	0.1841 (3)	1.0304 (3)	0.6278 (3)	0.0127 (7)	
C27	0.2436 (3)	1.0412 (3)	0.7085 (3)	0.0122 (7)	
H27	0.2003	1.0924	0.7300	0.015*	
C28	0.3695 (3)	1.0996 (3)	0.6531 (3)	0.0129 (7)	
C29	0.4198 (3)	1.1257 (3)	0.7329 (3)	0.0163 (7)	
H29A	0.4215	1.2063	0.7082	0.020*	
H29B	0.3731	1.0822	0.8120	0.020*	
C30	0.5411 (3)	1.0923 (3)	0.7248 (4)	0.0271 (9)	
H30A	0.5963	1.1539	0.6596	0.033*	
H30B	0.5658	1.0714	0.7954	0.033*	
C31	0.5307 (3)	0.9926 (3)	0.7078 (4)	0.0279 (9)	
H31A	0.4947	0.9244	0.7803	0.033*	
H31B	0.6067	0.9788	0.6758	0.033*	
C32	0.4547 (3)	1.0274 (3)	0.6243 (3)	0.0167 (8)	
H32A	0.4128	0.9608	0.6331	0.020*	
H32B	0.5013	1.0714	0.5452	0.020*	
C33	0.2289 (3)	0.9305 (3)	0.8186 (3)	0.0127 (7)	
C34	0.1865 (3)	0.9270 (3)	0.9199 (3)	0.0165 (8)	
C35	0.1772 (3)	0.8260 (3)	1.0245 (3)	0.0206 (8)	
C36	0.1474 (3)	1.0222 (3)	0.9246 (3)	0.0193 (8)	
C37	0.1739 (3)	1.0779 (3)	0.4380 (3)	0.0162 (7)	
C38	0.2760 (3)	1.2720 (3)	0.5729 (3)	0.0150 (7)	
S1	0.5191 (4)	0.2016 (3)	0.9487 (3)	0.0527 (7)	0.50
O1	0.3269 (6)	0.0090 (5)	1.0456 (5)	0.0475 (17)	0.50
C39	0.3016 (8)	0.0856 (6)	1.0644 (6)	0.038 (2)	0.50
H39	0.2264	0.0802	1.1067	0.046*	0.50
C40	0.3831 (6)	0.1866 (7)	1.0241 (5)	0.038 (2)	0.50
C41	0.3604 (14)	0.2732 (10)	1.0424 (10)	0.0546 (8)	0.50
H41	0.2887	0.2782	1.0833	0.066*	0.50
C42	0.4531 (9)	0.3522 (9)	0.9949 (8)	0.065 (3)	0.50
H42	0.4536	0.4191	0.9987	0.078*	0.50
C43	0.5438 (10)	0.3246 (6)	0.9423 (7)	0.054 (3)	0.50
H43	0.6154	0.3704	0.9048	0.064*	0.50
S1'	0.3563 (4)	0.2909 (3)	1.0178 (3)	0.0546 (8)	0.50
O1'	0.5647 (7)	0.4697 (6)	0.9167 (6)	0.072 (2)	0.50
C39'	0.5830 (9)	0.3837 (7)	0.9125 (7)	0.053 (3)	0.50
H39'	0.6586	0.3787	0.8789	0.063*	0.50
C40'	0.4948 (6)	0.2880 (8)	0.9564 (5)	0.041 (3)	0.50
C41'	0.5067 (14)	0.1919 (10)	0.9553 (9)	0.0527 (7)	0.50
H41'	0.5776	0.1758	0.9243	0.063*	0.50
C42'	0.4095 (8)	0.1200 (8)	1.0020 (7)	0.044 (2)	0.50
H42'	0.4042	0.0486	1.0078	0.053*	0.50
C43'	0.3215 (9)	0.1629 (7)	1.0391 (6)	0.051 (3)	0.50
H43'	0.2462	0.1243	1.0744	0.061*	0.50

### Atomic displacement parameters (Å^2^)

**Table:**

	*U*^11^	*U*^22^	*U*^33^	*U*^12^	*U*^13^	*U*^23^
N1	0.0205 (16)	0.0246 (16)	0.0219 (19)	0.0060 (13)	−0.0030 (14)	−0.0118 (15)
N2	0.0155 (15)	0.0125 (14)	0.0216 (18)	0.0030 (11)	−0.0023 (13)	−0.0048 (13)
N3	0.0218 (16)	0.0111 (14)	0.0191 (18)	0.0012 (11)	−0.0015 (13)	−0.0054 (13)
N4	0.0316 (18)	0.0172 (16)	0.0164 (18)	−0.0003 (13)	−0.0026 (14)	−0.0019 (14)
N5	0.040 (2)	0.0175 (16)	0.0218 (19)	0.0057 (14)	−0.0104 (15)	−0.0088 (14)
N6	0.0261 (17)	0.0133 (14)	0.0215 (18)	0.0028 (12)	−0.0055 (14)	−0.0077 (14)
N7	0.0260 (17)	0.0238 (16)	0.0239 (19)	0.0025 (13)	−0.0096 (14)	−0.0136 (15)
N8	0.0219 (15)	0.0101 (13)	0.0157 (17)	0.0016 (11)	−0.0090 (13)	−0.0032 (12)
N9	0.0236 (16)	0.0116 (13)	0.0129 (16)	0.0024 (11)	−0.0070 (13)	−0.0035 (12)
N10	0.044 (2)	0.0232 (17)	0.021 (2)	0.0066 (15)	−0.0112 (16)	−0.0067 (16)
N11	0.041 (2)	0.0181 (16)	0.0217 (19)	0.0016 (14)	−0.0001 (15)	−0.0093 (15)
N12	0.0275 (17)	0.0113 (14)	0.0208 (18)	0.0018 (12)	−0.0070 (14)	−0.0074 (13)
C1	0.0172 (16)	0.0097 (15)	0.0162 (19)	0.0040 (12)	−0.0050 (14)	−0.0075 (14)
C2	0.0199 (18)	0.0136 (16)	0.020 (2)	0.0069 (13)	−0.0064 (15)	−0.0068 (15)
C3	0.032 (2)	0.0210 (19)	0.018 (2)	0.0074 (16)	−0.0072 (17)	−0.0074 (17)
C4	0.0238 (19)	0.0185 (17)	0.015 (2)	0.0029 (14)	−0.0045 (15)	−0.0073 (16)
C5	0.0164 (16)	0.0097 (15)	0.0152 (19)	0.0023 (12)	−0.0046 (14)	−0.0065 (14)
C6	0.0151 (16)	0.0092 (15)	0.0140 (19)	0.0028 (12)	−0.0038 (14)	−0.0045 (14)
C7	0.0159 (16)	0.0080 (15)	0.017 (2)	0.0014 (12)	−0.0044 (14)	−0.0055 (14)
C8	0.0177 (17)	0.0118 (15)	0.0130 (19)	0.0021 (13)	−0.0034 (14)	−0.0064 (14)
C9	0.0178 (17)	0.0109 (15)	0.0147 (19)	0.0039 (13)	−0.0057 (14)	−0.0047 (14)
C10	0.0205 (18)	0.0126 (16)	0.018 (2)	0.0042 (13)	−0.0078 (15)	−0.0081 (15)
C11	0.0220 (19)	0.034 (2)	0.027 (2)	−0.0009 (17)	−0.0042 (17)	−0.0133 (19)
C12	0.028 (2)	0.038 (2)	0.033 (3)	−0.0014 (18)	0.0011 (19)	−0.021 (2)
C13	0.0214 (18)	0.0168 (17)	0.016 (2)	0.0073 (14)	−0.0045 (15)	−0.0105 (15)
C14	0.0116 (16)	0.0140 (16)	0.018 (2)	0.0035 (12)	−0.0030 (14)	−0.0071 (15)
C15	0.0217 (18)	0.0112 (16)	0.016 (2)	0.0033 (13)	−0.0061 (15)	−0.0047 (15)
C16	0.0219 (19)	0.0192 (18)	0.021 (2)	0.0046 (15)	−0.0031 (16)	−0.0114 (17)
C17	0.0229 (19)	0.0200 (18)	0.016 (2)	0.0070 (15)	−0.0034 (15)	−0.0103 (16)
C18	0.0185 (17)	0.0107 (15)	0.0159 (19)	0.0019 (13)	−0.0052 (15)	−0.0048 (14)
C19	0.0197 (17)	0.0080 (15)	0.015 (2)	0.0033 (13)	−0.0008 (14)	−0.0039 (14)
C20	0.0166 (16)	0.0078 (14)	0.0142 (19)	0.0010 (12)	−0.0061 (14)	−0.0036 (14)
C21	0.0202 (18)	0.0141 (16)	0.017 (2)	−0.0029 (13)	−0.0049 (15)	−0.0054 (15)
C22	0.032 (2)	0.0214 (19)	0.014 (2)	−0.0091 (16)	0.0002 (16)	−0.0038 (16)
C23	0.0273 (19)	0.0185 (18)	0.014 (2)	−0.0028 (15)	−0.0043 (15)	−0.0073 (16)
C24	0.0173 (17)	0.0136 (16)	0.0158 (19)	0.0019 (13)	−0.0047 (14)	−0.0093 (15)
C25	0.0166 (16)	0.0105 (15)	0.0159 (19)	0.0023 (13)	−0.0070 (14)	−0.0076 (14)
C26	0.0150 (16)	0.0087 (15)	0.0161 (19)	0.0054 (12)	−0.0071 (14)	−0.0063 (14)
C27	0.0159 (16)	0.0080 (14)	0.0109 (18)	0.0023 (12)	−0.0046 (13)	−0.0029 (14)
C28	0.0177 (17)	0.0083 (15)	0.0122 (18)	0.0006 (12)	−0.0046 (14)	−0.0042 (14)
C29	0.0191 (17)	0.0159 (17)	0.0139 (19)	0.0000 (13)	−0.0068 (14)	−0.0065 (15)
C30	0.024 (2)	0.028 (2)	0.034 (2)	0.0049 (16)	−0.0169 (18)	−0.0156 (19)
C31	0.028 (2)	0.025 (2)	0.036 (3)	0.0131 (16)	−0.0181 (19)	−0.0155 (19)
C32	0.0166 (17)	0.0118 (16)	0.019 (2)	0.0024 (13)	−0.0056 (15)	−0.0050 (15)
C33	0.0142 (16)	0.0095 (15)	0.0110 (18)	−0.0011 (12)	−0.0047 (13)	−0.0019 (14)
C34	0.0224 (18)	0.0137 (16)	0.0127 (19)	−0.0012 (13)	−0.0056 (15)	−0.0054 (15)
C35	0.029 (2)	0.0166 (18)	0.019 (2)	0.0004 (15)	−0.0079 (16)	−0.0096 (17)
C36	0.0255 (19)	0.0165 (17)	0.0100 (19)	−0.0024 (14)	−0.0007 (15)	−0.0036 (15)
C37	0.0202 (17)	0.0107 (15)	0.0155 (19)	0.0021 (13)	−0.0051 (15)	−0.0045 (14)
C38	0.0218 (18)	0.0091 (15)	0.016 (2)	0.0005 (13)	−0.0096 (15)	−0.0061 (15)
S1	0.0638 (17)	0.0479 (14)	0.0463 (14)	0.0097 (11)	−0.0051 (12)	−0.0267 (11)
O1	0.060 (4)	0.040 (3)	0.054 (4)	0.014 (3)	−0.027 (3)	−0.026 (3)
C39	0.041 (5)	0.043 (5)	0.032 (5)	0.007 (4)	−0.020 (4)	−0.016 (4)
C40	0.050 (6)	0.035 (5)	0.038 (5)	0.011 (4)	−0.021 (4)	−0.021 (4)
C41	0.0418 (12)	0.0609 (18)	0.077 (2)	0.0194 (12)	−0.0207 (14)	−0.0436 (17)
C42	0.070 (7)	0.052 (6)	0.083 (7)	0.003 (5)	−0.023 (6)	−0.040 (5)
C43	0.051 (7)	0.044 (6)	0.062 (7)	−0.014 (5)	−0.001 (5)	−0.029 (5)
S1'	0.0418 (12)	0.0609 (18)	0.077 (2)	0.0194 (12)	−0.0207 (14)	−0.0436 (17)
O1'	0.074 (5)	0.054 (4)	0.080 (5)	0.013 (4)	−0.010 (4)	−0.031 (4)
C39'	0.047 (6)	0.053 (6)	0.056 (6)	0.019 (5)	−0.016 (5)	−0.023 (5)
C40'	0.039 (5)	0.042 (5)	0.037 (5)	0.008 (4)	−0.014 (4)	−0.015 (4)
C41'	0.0638 (17)	0.0479 (14)	0.0463 (14)	0.0097 (11)	−0.0051 (12)	−0.0267 (11)
C42'	0.058 (6)	0.050 (5)	0.034 (5)	0.007 (4)	−0.021 (4)	−0.024 (4)
C43'	0.057 (6)	0.050 (6)	0.055 (6)	0.010 (5)	−0.027 (5)	−0.029 (5)

### Geometric parameters (Å, °)

**Table:**

N1—C18	1.152 (4)	C20—C24	1.515 (5)
N2—C7	1.349 (4)	C20—C21	1.555 (4)
N2—H2N1	0.8600	C20—C28	1.565 (4)
N2—H2N2	0.8600	C21—C22	1.538 (5)
N3—C14	1.327 (4)	C21—H21A	0.9900
N3—H3N1	0.8600	C21—H21B	0.9900
N3—H3N2	0.8600	C22—C23	1.499 (5)
N4—C16	1.144 (4)	C22—H22A	0.9900
N5—C17	1.148 (4)	C22—H22B	0.9900
N6—C19	1.149 (4)	C23—C24	1.328 (5)
N7—C37	1.152 (4)	C23—H23	0.9500
N8—C26	1.336 (4)	C24—C25	1.456 (4)
N8—H8N1	0.8600	C25—C26	1.369 (5)
N8—H8N2	0.8600	C25—C37	1.425 (5)
N9—C33	1.335 (4)	C26—C27	1.514 (5)
N9—H9N1	0.8600	C27—C33	1.519 (4)
N9—H9N2	0.8600	C27—C28	1.562 (4)
N10—C35	1.148 (5)	C27—H27	1.0000
N11—C36	1.159 (5)	C28—C29	1.550 (5)
N12—C38	1.145 (4)	C28—C32	1.554 (5)
C1—C19	1.490 (5)	C29—C30	1.526 (5)
C1—C5	1.514 (5)	C29—H29A	0.9900
C1—C9	1.548 (4)	C29—H29B	0.9900
C1—C2	1.557 (5)	C30—C31	1.516 (5)
C2—C3	1.539 (5)	C30—H30A	0.9900
C2—H2A	0.9900	C30—H30B	0.9900
C2—H2B	0.9900	C31—C32	1.520 (5)
C3—C4	1.492 (5)	C31—H31A	0.9900
C3—H3A	0.9900	C31—H31B	0.9900
C3—H3B	0.9900	C32—H32A	0.9900
C4—C5	1.332 (5)	C32—H32B	0.9900
C4—H4	0.9500	C33—C34	1.370 (5)
C5—C6	1.447 (5)	C34—C35	1.421 (5)
C6—C7	1.368 (5)	C34—C36	1.422 (5)
C6—C18	1.414 (5)	S1—C43	1.681 (8)
C7—C8	1.505 (5)	S1—C40	1.698 (8)
C8—C14	1.522 (4)	O1—C39	1.231 (8)
C8—C9	1.569 (5)	C39—C40	1.492 (8)
C8—H8	1.0000	C39—H39	0.9500
C9—C13	1.542 (5)	C40—C41	1.355 (10)
C9—C10	1.564 (4)	C41—C42	1.364 (10)
C10—C11	1.530 (5)	C41—H41	0.9500
C10—H10A	0.9900	C42—C43	1.343 (9)
C10—H10B	0.9900	C42—H42	0.9500
C11—C12	1.502 (6)	C43—H43	0.9500
C11—H11A	0.9900	S1'—C43'	1.682 (8)
C11—H11B	0.9900	S1'—C40'	1.714 (8)
C12—C13	1.526 (5)	O1'—C39'	1.234 (8)
C12—H12A	0.9900	C39'—C40'	1.479 (8)
C12—H12B	0.9900	C39'—H39'	0.9500
C13—H13A	0.9900	C40'—C41'	1.340 (10)
C13—H13B	0.9900	C41'—C42'	1.351 (10)
C14—C15	1.378 (5)	C41'—H41'	0.9500
C15—C17	1.423 (5)	C42'—C43'	1.342 (9)
C15—C16	1.430 (5)	C42'—H42'	0.9500
C20—C38	1.478 (5)	C43'—H43'	0.9500
			
C7—N2—H2N1	109.5	C20—C21—H21B	110.7
C7—N2—H2N2	109.5	H21A—C21—H21B	108.8
H2N1—N2—H2N2	109.5	C23—C22—C21	104.2 (3)
C14—N3—H3N1	109.5	C23—C22—H22A	110.9
C14—N3—H3N2	109.5	C21—C22—H22A	110.9
H3N1—N3—H3N2	109.5	C23—C22—H22B	110.9
C26—N8—H8N1	109.5	C21—C22—H22B	110.9
C26—N8—H8N2	109.5	H22A—C22—H22B	108.9
H8N1—N8—H8N2	109.5	C24—C23—C22	111.9 (3)
C33—N9—H9N1	109.5	C24—C23—H23	124.1
C33—N9—H9N2	109.5	C22—C23—H23	124.1
H9N1—N9—H9N2	109.5	C23—C24—C25	132.4 (3)
C19—C1—C5	107.9 (3)	C23—C24—C20	112.1 (3)
C19—C1—C9	107.8 (3)	C25—C24—C20	115.4 (3)
C5—C1—C9	110.4 (3)	C26—C25—C37	119.9 (3)
C19—C1—C2	110.7 (3)	C26—C25—C24	122.7 (3)
C5—C1—C2	103.0 (3)	C37—C25—C24	117.3 (3)
C9—C1—C2	116.8 (3)	N8—C26—C25	123.0 (3)
C3—C2—C1	105.4 (3)	N8—C26—C27	116.3 (3)
C3—C2—H2A	110.7	C25—C26—C27	120.5 (3)
C1—C2—H2A	110.7	C26—C27—C33	110.9 (3)
C3—C2—H2B	110.7	C26—C27—C28	114.1 (3)
C1—C2—H2B	110.7	C33—C27—C28	114.6 (3)
H2A—C2—H2B	108.8	C26—C27—H27	105.4
C4—C3—C2	103.9 (3)	C33—C27—H27	105.4
C4—C3—H3A	111.0	C28—C27—H27	105.4
C2—C3—H3A	111.0	C29—C28—C32	104.7 (3)
C4—C3—H3B	111.0	C29—C28—C27	112.1 (3)
C2—C3—H3B	111.0	C32—C28—C27	113.7 (2)
H3A—C3—H3B	109.0	C29—C28—C20	111.3 (2)
C5—C4—C3	112.5 (3)	C32—C28—C20	110.4 (3)
C5—C4—H4	123.8	C27—C28—C20	104.8 (3)
C3—C4—H4	123.8	C30—C29—C28	105.8 (3)
C4—C5—C6	131.9 (3)	C30—C29—H29A	110.6
C4—C5—C1	111.7 (3)	C28—C29—H29A	110.6
C6—C5—C1	116.2 (3)	C30—C29—H29B	110.6
C7—C6—C18	119.9 (3)	C28—C29—H29B	110.6
C7—C6—C5	121.5 (3)	H29A—C29—H29B	108.7
C18—C6—C5	118.2 (3)	C31—C30—C29	103.4 (3)
N2—C7—C6	123.9 (3)	C31—C30—H30A	111.1
N2—C7—C8	115.4 (3)	C29—C30—H30A	111.1
C6—C7—C8	120.6 (3)	C31—C30—H30B	111.1
C7—C8—C14	109.5 (3)	C29—C30—H30B	111.1
C7—C8—C9	116.0 (3)	H30A—C30—H30B	109.0
C14—C8—C9	114.8 (3)	C30—C31—C32	103.3 (3)
C7—C8—H8	105.1	C30—C31—H31A	111.1
C14—C8—H8	105.1	C32—C31—H31A	111.1
C9—C8—H8	105.1	C30—C31—H31B	111.1
C13—C9—C1	112.5 (3)	C32—C31—H31B	111.1
C13—C9—C10	104.9 (3)	H31A—C31—H31B	109.1
C1—C9—C10	109.4 (3)	C31—C32—C28	105.9 (3)
C13—C9—C8	111.2 (3)	C31—C32—H32A	110.6
C1—C9—C8	104.7 (3)	C28—C32—H32A	110.6
C10—C9—C8	114.3 (3)	C31—C32—H32B	110.6
C11—C10—C9	105.6 (3)	C28—C32—H32B	110.6
C11—C10—H10A	110.6	H32A—C32—H32B	108.7
C9—C10—H10A	110.6	N9—C33—C34	122.3 (3)
C11—C10—H10B	110.6	N9—C33—C27	118.5 (3)
C9—C10—H10B	110.6	C34—C33—C27	119.2 (3)
H10A—C10—H10B	108.8	C33—C34—C35	120.5 (3)
C12—C11—C10	104.8 (3)	C33—C34—C36	121.7 (3)
C12—C11—H11A	110.8	C35—C34—C36	117.8 (3)
C10—C11—H11A	110.8	N10—C35—C34	177.5 (4)
C12—C11—H11B	110.8	N11—C36—C34	178.6 (4)
C10—C11—H11B	110.8	N7—C37—C25	179.3 (4)
H11A—C11—H11B	108.9	N12—C38—C20	177.2 (3)
C11—C12—C13	103.6 (3)	C43—S1—C40	89.2 (5)
C11—C12—H12A	111.0	O1—C39—C40	123.4 (9)
C13—C12—H12A	111.0	O1—C39—H39	118.3
C11—C12—H12B	111.0	C40—C39—H39	118.3
C13—C12—H12B	111.0	C41—C40—C39	126.1 (9)
H12A—C12—H12B	109.0	C41—C40—S1	113.0 (9)
C12—C13—C9	105.6 (3)	C39—C40—S1	120.9 (7)
C12—C13—H13A	110.6	C40—C41—C42	111.7 (13)
C9—C13—H13A	110.6	C40—C41—H41	124.1
C12—C13—H13B	110.6	C42—C41—H41	124.1
C9—C13—H13B	110.6	C43—C42—C41	112.2 (12)
H13A—C13—H13B	108.8	C43—C42—H42	123.9
N3—C14—C15	122.2 (3)	C41—C42—H42	123.9
N3—C14—C8	117.8 (3)	C42—C43—S1	113.9 (8)
C15—C14—C8	119.9 (3)	C42—C43—H43	123.1
C14—C15—C17	121.6 (3)	S1—C43—H43	123.1
C14—C15—C16	119.6 (3)	C43'—S1'—C40'	89.4 (5)
C17—C15—C16	118.6 (3)	O1'—C39'—C40'	124.3 (10)
N4—C16—C15	175.1 (4)	O1'—C39'—H39'	117.9
N5—C17—C15	177.1 (4)	C40'—C39'—H39'	117.9
N1—C18—C6	179.4 (4)	C41'—C40'—C39'	128.4 (9)
N6—C19—C1	176.7 (4)	C41'—C40'—S1'	111.0 (10)
C38—C20—C24	108.4 (3)	C39'—C40'—S1'	120.6 (8)
C38—C20—C21	109.8 (3)	C40'—C41'—C42'	114.6 (14)
C24—C20—C21	102.9 (3)	C40'—C41'—H41'	122.7
C38—C20—C28	108.4 (3)	C42'—C41'—H41'	122.7
C24—C20—C28	109.8 (2)	C43'—C42'—C41'	110.9 (12)
C21—C20—C28	117.2 (3)	C43'—C42'—H42'	124.5
C22—C21—C20	105.1 (3)	C41'—C42'—H42'	124.5
C22—C21—H21A	110.7	C42'—C43'—S1'	114.1 (9)
C20—C21—H21A	110.7	C42'—C43'—H43'	123.0
C22—C21—H21B	110.7	S1'—C43'—H43'	123.0
			
C19—C1—C2—C3	97.7 (3)	C38—C20—C24—C25	−72.8 (3)
C5—C1—C2—C3	−17.4 (3)	C21—C20—C24—C25	170.9 (3)
C9—C1—C2—C3	−138.5 (3)	C28—C20—C24—C25	45.5 (4)
C1—C2—C3—C4	18.0 (3)	C23—C24—C25—C26	175.6 (4)
C2—C3—C4—C5	−12.2 (4)	C20—C24—C25—C26	−8.6 (5)
C3—C4—C5—C6	−175.7 (3)	C23—C24—C25—C37	−7.9 (6)
C3—C4—C5—C1	0.8 (4)	C20—C24—C25—C37	167.9 (3)
C19—C1—C5—C4	−106.3 (3)	C37—C25—C26—N8	−8.1 (5)
C9—C1—C5—C4	136.1 (3)	C24—C25—C26—N8	168.3 (3)
C2—C1—C5—C4	10.7 (4)	C37—C25—C26—C27	176.9 (3)
C19—C1—C5—C6	70.8 (3)	C24—C25—C26—C27	−6.6 (5)
C9—C1—C5—C6	−46.8 (4)	N8—C26—C27—C33	37.1 (4)
C2—C1—C5—C6	−172.1 (3)	C25—C26—C27—C33	−147.6 (3)
C4—C5—C6—C7	−174.7 (3)	N8—C26—C27—C28	168.3 (3)
C1—C5—C6—C7	8.9 (4)	C25—C26—C27—C28	−16.4 (4)
C4—C5—C6—C18	11.5 (5)	C26—C27—C28—C29	170.8 (3)
C1—C5—C6—C18	−164.9 (3)	C33—C27—C28—C29	−59.8 (4)
C18—C6—C7—N2	6.0 (5)	C26—C27—C28—C32	−70.7 (4)
C5—C6—C7—N2	−167.6 (3)	C33—C27—C28—C32	58.7 (4)
C18—C6—C7—C8	−176.8 (3)	C26—C27—C28—C20	49.9 (3)
C5—C6—C7—C8	9.5 (4)	C33—C27—C28—C20	179.3 (3)
N2—C7—C8—C14	−39.7 (4)	C38—C20—C28—C29	−67.2 (3)
C6—C7—C8—C14	142.9 (3)	C24—C20—C28—C29	174.5 (3)
N2—C7—C8—C9	−171.6 (3)	C21—C20—C28—C29	57.7 (4)
C6—C7—C8—C9	11.0 (4)	C38—C20—C28—C32	177.0 (2)
C19—C1—C9—C13	65.2 (3)	C24—C20—C28—C32	58.7 (3)
C5—C1—C9—C13	−177.2 (3)	C21—C20—C28—C32	−58.1 (4)
C2—C1—C9—C13	−60.1 (4)	C38—C20—C28—C27	54.2 (3)
C19—C1—C9—C10	−178.6 (3)	C24—C20—C28—C27	−64.1 (3)
C5—C1—C9—C10	−61.0 (3)	C21—C20—C28—C27	179.1 (3)
C2—C1—C9—C10	56.1 (4)	C32—C28—C29—C30	12.5 (3)
C19—C1—C9—C8	−55.7 (3)	C27—C28—C29—C30	136.3 (3)
C5—C1—C9—C8	61.9 (3)	C20—C28—C29—C30	−106.7 (3)
C2—C1—C9—C8	179.0 (3)	C28—C29—C30—C31	−33.5 (4)
C7—C8—C9—C13	−167.1 (3)	C29—C30—C31—C32	41.6 (4)
C14—C8—C9—C13	63.5 (3)	C30—C31—C32—C28	−33.9 (4)
C7—C8—C9—C1	−45.3 (3)	C29—C28—C32—C31	13.1 (3)
C14—C8—C9—C1	−174.7 (3)	C27—C28—C32—C31	−109.6 (3)
C7—C8—C9—C10	74.4 (3)	C20—C28—C32—C31	133.0 (3)
C14—C8—C9—C10	−55.0 (4)	C26—C27—C33—N9	52.2 (4)
C13—C9—C10—C11	6.0 (4)	C28—C27—C33—N9	−78.8 (4)
C1—C9—C10—C11	−114.9 (3)	C26—C27—C33—C34	−126.9 (3)
C8—C9—C10—C11	128.0 (3)	C28—C27—C33—C34	102.1 (4)
C9—C10—C11—C12	−28.3 (4)	N9—C33—C34—C35	4.2 (5)
C10—C11—C12—C13	39.6 (4)	C27—C33—C34—C35	−176.8 (3)
C11—C12—C13—C9	−35.8 (4)	N9—C33—C34—C36	−173.9 (3)
C1—C9—C13—C12	136.9 (3)	C27—C33—C34—C36	5.1 (5)
C10—C9—C13—C12	18.0 (4)	C28—C20—C38—N12	−52 (8)
C8—C9—C13—C12	−106.0 (3)	O1—C39—C40—C41	−179.8 (2)
C7—C8—C14—N3	−50.7 (4)	O1—C39—C40—S1	−0.3 (3)
C9—C8—C14—N3	81.8 (4)	C43—S1—C40—C41	−0.2 (2)
C7—C8—C14—C15	127.3 (3)	C43—S1—C40—C39	−179.7 (2)
C9—C8—C14—C15	−100.2 (4)	C39—C40—C41—C42	179.7 (2)
N3—C14—C15—C17	171.0 (3)	S1—C40—C41—C42	0.2 (3)
C8—C14—C15—C17	−6.9 (5)	C40—C41—C42—C43	−0.2 (4)
N3—C14—C15—C16	−3.5 (5)	C41—C42—C43—S1	0.1 (4)
C8—C14—C15—C16	178.5 (3)	C40—S1—C43—C42	0.1 (2)
C38—C20—C21—C22	−96.4 (3)	O1'—C39'—C40'—C41'	179.9 (2)
C24—C20—C21—C22	18.8 (4)	O1'—C39'—C40'—S1'	0.2 (3)
C28—C20—C21—C22	139.4 (3)	C43'—S1'—C40'—C41'	0.1 (2)
C20—C21—C22—C23	−18.8 (4)	C43'—S1'—C40'—C39'	179.8 (2)
C21—C22—C23—C24	12.0 (4)	C39'—C40'—C41'—C42'	−179.9 (2)
C22—C23—C24—C25	176.3 (4)	S1'—C40'—C41'—C42'	−0.2 (4)
C22—C23—C24—C20	0.4 (4)	C40'—C41'—C42'—C43'	0.2 (4)
C38—C20—C24—C23	103.8 (3)	C41'—C42'—C43'—S1'	−0.1 (3)
C21—C20—C24—C23	−12.4 (4)	C40'—S1'—C43'—C42'	0.0 (2)
C28—C20—C24—C23	−137.9 (3)		

### Hydrogen-bond geometry (Å, °)

**Table:**

*D*—H···*A*	*D*—H	H···*A*	*D*···*A*	*D*—H···*A*
N2—H2n1···N1^i^	0.86	2.23	2.909 (4)	136
N3—H3n1···N11^ii^	0.86	2.28	2.970 (4)	138
N3—H3n2···N12^ii^	0.86	2.29	3.042 (4)	147
N8—H8n1···N7^iii^	0.86	2.45	2.921 (4)	115
N8—H8n2···N4^iv^	0.86	2.31	3.006 (4)	138
N9—H9n1···N5	0.86	2.39	3.098 (4)	139
N9—H9n2···N6	0.86	2.35	3.178 (4)	163

Symmetry codes: (i) −*x*+1, −*y*+1, −*z*+1; (ii) *x*, *y*−1, *z*; (iii) −*x*, −*y*+2, −*z*+1; (iv) −*x*, −*y*+1, −*z*+2.
